# The association between tissue doppler-derived E/(e′s′) ratio and coronary atherosclerosis severity measured by the SYNTAX score in patients with non-ST elevation-acute coronary syndrome

**DOI:** 10.1186/s12872-023-03128-8

**Published:** 2023-02-22

**Authors:** Maryam Nabati, Samad Golshani, Morteza Taghavi, Abbas Alipour, Homa Parsaee

**Affiliations:** 1 Department of Cardiology, Faculty of Medicine, Cardiovascular Research Center, Mazandaran University of Medical Sciences, Sari, Iran; 2 Faculty of Medicine, Cardiovascular Research Center, Mazandaran University of Medical Sciences, Sari, Iran; 3 Community Medicine Department, Medical Faculty, Mazandaran University of Medical Sciences, Sari, Iran; 4 Faculty of Medicine, Iran University of Medical Sciences, Tehran, Iran

**Keywords:** Diastolic dysfunction, Mitral annulus velocity, Non-ST elevation acute coronary syndrome, Systolic dysfunction, Tissue Doppler imaging

## Abstract

**Background:**

The prognosis of patients hospitalized with non-ST elevation-acute coronary syndrome (NSTE-ACS) is typically determined by the existence and severity of myocardial necrosis and left ventricular (LV) remodeling.

**Aim:**

The present study was to assess the association between the E/(e′s′) ratio and the coronary atherosclerosis severity, measured by the SYNTAX score, in patients with NSTE-ACS.

**Methods:**

Utilizing a descriptive correlational research design, this study was prospectively conducted on 252 patients with NSTE-ACS, undergoing an echocardiography to determine the left ventricular ejection fraction (LVEF), the left atrial (LA) volume, the pulsed-wave (PW) Doppler-derived transmitral early (E) and late (A) diastolic velocities, and the tissue Doppler (TD)-derived mitral annular early diastolic (e′) and peak systolic (s′) velocities. After that, a coronary angiography (CAG) was performed, and the SYNTAX score was calculated.

**Results:**

The patients were divided into two groups, viz., those with the E/(e′s′) ratio < 1.63 and the cases with the ratio ≥ 1.63. The results revealed that the patients with a high ratio were older, had a higher prevalence of females, a SYNTAX score ≥ 22, and a lower glomerular filtration rate than those with a low ratio (*p*-value < 0.001). Besides, these patients had larger indexed LA volume and lower LVEF than others (*p*-value: 0.028 and 0.023, respectively). Furthermore, the multiple linear regression outcomes established a positive independent association between the E/(e′s′) ratio ≥ 1.63 (B = 5.609, 95% CI 2.324–8.894, *p*-value = 0.001) and the SYNTAX score.

**Conclusion:**

The study results demonstrated that the patients hospitalized with NSTE-ACS and the E/(e′s′) ratio ≥ 1.63 had worse demographic, echocardiographic, and laboratory profiles and a higher prevalence of a SYNTAX score ≥ 22 than those with a lower ratio.

## Introduction

Almost half of the cases with cardiovascular disease (CVD) mortality are due to non-ST elevation-acute coronary syndrome (NSTE-ACS). Risk stratification is thus of utmost importance to identify patients at high risk for adverse outcomes and even death, as they may benefit from an invasive treatment strategy to minimize the complications [1]. In this respect, the Global Registry of Acute Coronary Events (GRACE) risk score, as a scoring system, has been validated for the assessment of in-hospital and one- and 12-month mortality. Higher GRACE score is accordingly associated with more severe angiographic characteristics of coronary artery disease (CAD) and worse prognosis, so the score > 140 indicates high morbidity and mortality rates [2]. On the other hand, the acute coronary syndrome (ACS) guidelines have recommended performing an echocardiography in the emergency department to aid in the differential diagnosis of the chest pain syndrome, and then stratify the risk of patients with this condition. Many echocardiographic parameters, such as the left ventricular ejection fraction (LVEF), the early transmitral flow velocity to the early diastolic mitral annulus velocity (E/e′) ratio, the mitral inflow E-wave deceleration time, and the mitral regurgitation (MR) have been thus linked to the outcomes in patients with ACS [3]. Indeed, the prognosis of patients with NSTE-ACS is typically determined by the existence and severity of myocardial necrosis and left ventricular (LV) remodeling [4]. In this way, some patients may be presented with asymptomatic functional or structural LV dysfunction before the development of symptomatic heart failure (HF) [5]. Ischemia can further multiply the LV filling pressure by impairing myocardial contractility and relaxation [6]. It also seems that tissue Doppler (TD) imaging is a useful tool for measuring an adverse outcome in several CVDs. A significant drop in the TD-derived mitral annular peak systolic (s′) and early diastolic (e′) velocities has been already detected in patients with ischemic heart disease (IHD) [7]. Previous research has further established that an e′ velocity and an E/e′ ratio can be among the strong predictors of the adverse events following acute myocardial infarction (MI) [8]. An s′ wave also reflects the peak systolic velocity of the longitudinally-orientated myocardial fibers in the sub-endocardial region, which are mostly susceptible to ischemic injury. An s′ velocity may be accordingly a more important predictor of outcomes than the LVEF. It is due to the fact that an s′ velocity can be a sign of subclinical LV systolic dysfunction. A new TD index, namely, the E/(e′s′) ratio, has been recently proposed, which simultaneously incorporates the parameters of the diastolic (E/e′) and systolic (s′) functions. Although little research has so far investigated the prognostic role of E/(e′s′) in patients with NSTE-ACS, a recent study showed that cases with NSTE-ACS and an initial E/(e′s′) ratio > 1.63 had the worst prognosis regarding the composite outcomes, readmissions, and cardiac deaths [1]. On the other hand, the SYNTAX score has been introduced as an angiographic scoring system that helps combine multiple entities, including the extent, complexity, and severity of CAD [9]. However, SYNTAX score is not able to combine clinical variables with coronary anatomy. Therefore, SYNTAX Score II (SSII) has been developed and was a combination of two anatomical and six clinical variables [10]. Previous studies have further reported that a higher SYNTAX score often indicates a more complex lesion and an unfavorable prognosis, so it is a strong independent predictor of both in-hospital and long-term mortality rates in patients with acute MI [9, 11]. Also, SSII is predictive of long-term clinical outcome and 4-year mortality [10]. The electrocardiographic diastolic index is a simple and inexpensive formula which seems to play an important role in predicting diastolic dysfunction [12]. The risk factors for diastolic dysfunction and coronary artery disease are similar and a diastolic dysfunction scoring system including SYNTAX and echocardiography should be formed in order to determine these patients. Accordingly, the present study aimed to assess the association between the E/(e′s′) ratio and the SYNTAX score in patients with NSTE-ACS.

## Methodology

This study with a descriptive correlational design was conducted on 252 patients with NSTE-ACS, admitted to the emergency department of our hospital between 2021 and 2022. The method of data collection was longitudinally and prospectively designed over the time. All patients underwent a transthoracic echocardiography during the first day of hospitalization and the echocardiographic data were interpreted by one echocardiographer. Also, a coronary angiography (CAG) was performed for all patients during hospitalization and the SYNTAX score was computed by an experienced cardiologist to reduce the measurement errors and avoid human bias. The given patients were then divided into two groups based on their echocardiographic E/(e′s′) ratio, viz., those with the ratio < 1.63 and the individuals with the ratio ≥ 1.63, and the demographic, laboratory, and other echocardiographic variables and SYNTAX score were compared between two groups. This study was also fulfilled in accordance with the Declaration of Helsinki (DoH) guidelines, and approved by the ethics committee of the hospital. A signed informed consent was further obtained from all patients. Besides, ACS was diagnosed based on the 2020 European Society of Cardiology (ESC) guidelines for the management of acute coronary syndromes in patients presenting without persistent ST-segment elevation [13]. The patients with transient ST changes (≥ 0.5 mm) or deep T-wave inversion during angina symptoms and those with symptoms suggestive of ischemia, but without ST-T changes, were accordingly included in the study. NSTE-myocardial infarction (MI) was further diagnosed in keeping with the presence of angina and a transient rise in cardiac troponin I (TnI) levels above the upper normal limits (0.04 ng/mL). The TnI levels were measured within 10 min of presentation, and the measurements were repeated at the intervals of 4–6 h during the first two days. Unstable angina was then diagnosed when there was new-onset, at rest, or accelerating angina, and no rise in TnI above the upper normal limits [14]. The patients with persistent ST-segment elevation of 0.1 mv or more in leads other than aVR or V1, left bundle branch block (LBBB), anterior ST depression indicative of posterior ST-segment elevation MI, significant primary valvular heart disease, atrial fibrillation (AF), cardiomyopathies, a previous history of MI, coronary artery bypass graft (CABG), or percutaneous coronary intervention (PCI), acute HF, a history of recent chemotherapy, any systemic illnesses, fever, and pregnancy were excluded from the study. Of note, the patients were also excluded if they had chronic obstructive pulmonary disease (COPD) as well as emphysema or any known interstitial lung disease, because impaired lung function and hyperinflation were associated with a decrease in left heart preload and a small, but measurable, effect on diastolic variables [15]. All patients underwent coronary angiography (CAG) by a Siemens AG (Medical Solutions; Erlangen, Germany). An experienced cardiologist blinded to the patient data then interpreted the angiograms. The SYNTAX score was further implemented to determine the CAD extent, severity, and complexity. The patient’s overall SYNTAX score was also computed from the summation of these individual scores for each lesion by an algorithm of the SYNTAX score accessible on the website of SYNTAX (http://www.syntaxscore.com) [16]. The SYNTAX reproducibility was further determined based on repeated measurements in 25 randomly selected patients by a cardiologist within 48 h, and the intra-observer correlation coefficient was found to be 0.89.The patients were accordingly divided into two groups with high (≥ 22) and low (< 22) SYNTAX scores [17]. Hypertension (HTN) was additionally respected as the systolic blood pressure(SBP) ≥ 140 mmHg and/or the diastolic blood pressure (DBP) ≥ 90 mmHg or receiving antihypertensive drugs [18]. Diabetes mellitus (DM) was further diagnosed according to the American Diabetes Association (ADA) guidelines, and incorporated the patients in need of receiving insulin or oral hypoglycemic medications [19]. Likewise, hyperlipidemia (HLP) was defined as the total cholesterol (TC) levels of > 200 mg/dl, high-density lipoprotein cholesterol (LDL-c) levels < 40 mg/dl in males, or < 50 mg/dl in female cases [20]. The family history (FH) of CAD was then considered by the disease diagnosis in a male first-degree relative of less than 55 years or a female one under 65 [21]. The history of smoking was further determined by a face-to-face questionnaire. Moreover, body mass index (BMI) was computed as an individual’s weight (kilograms) divided by their height (meters) squared. The glomerular filtration rate (GFR) was then calculated by the following formula: GFR = (140-age) × (weight in kg)/72 × serum Cr (mg/dl) × 0.85 if female [22].

### Echocardiography

All patients underwent a transthoracic echocardiography by a Vivid S5 (GE Healthcare, Wauwatosa, WI) during the first day of hospitalization, and all images were stored on a hard disk. An experienced echocardiographer blinded to the patient data then interpreted them. The LVEF was further calculated by subtracting the LV end-systolic volume (LVESV) from the LV end-diastolic volume (LVEDV), divided by LVEDV by the biplane Simpson’s technique in both apical two- and four-chamber views. The left atrial (LA) volume was further determined by tracing the LA endocardial border in an apical four-chamber view [23]. Moreover, the transmitral Doppler early and late diastolic velocities (namely, the E and A waves) were acquired by inserting the cursor at the tip of the mitral valve leaflets in the apical four-chamber view. The average TD mitral annulus septal and lateral early diastolic (e′) and peak systolic (s′) velocities were correspondingly acquired by inserting the cursor at the level of mitral annulus [24]. The ratios of pulsed-wave (PW) Doppler E/A and TD E/e′ and E/(e′s′) were ultimately calculated. Previous studies had already shown that patients with NSTE-ACS and the initial E/(e′s′) ratio > 1.63 had the worst prognosis [1]. Therefore, the patients were divided into two groups, viz., the cases with the E/(e′s′) ratio < 1.63 and those with the ratio ≥ 1.63. The reproducibility of the TD E/(e′s′) ratio was further determined based on repeated measurements in 20 randomly selected patients by an echocardiographer within 24 h, and the intra-observer correlation coefficient was found to be 0.91.

### Statistical analysis

Based on the pilot study as well as the mean ± standard deviation (SD) of the difference between the E/e′ equal to 2.5 and 4 in the patients with the E/(e′s′) ratio < 1.63 and those with the ratio ≥ 1.63 and considering the power of the study by 90% and the probability of the type-I error equal to 0.01, a sample size of 76 patients was calculated. The prevalence of the patients with the E/(e′s′) ratio ≥ 1.63 was equal to 30%, and the total sample size was calculated as 252 patients.

The continuous variables were further explained as mean ± SD, and the categorical ones were illustrated as frequency and percentage. The Shapiro–Wilk test results also confirmed that all data were normally distributed. Therefore, the continuous variables were compared by an independent t*-*test and the categorical ones were measured using Chi-square and Fisher’s exact tests. Moreover, univariate linear regression (LR) analysis was employed to individually determine the relationship between different variables and the SYNTAX score. The variables with the *p*-value < 0.3 were also entered into multiple linear regression, using backward elimination. The *p*-value < 0.05 was further considered to be statistically significant. The statistical data were finally analyzed using the SPSS Statistics (version 18) software (SPSS, Chicago, IL, USA).

## Results

This study included 252 patients, admitted with NSTE-ACS. The mean age of the study population was 59.26 ± 9.98 years, and the mean BMI was 27.15 ± 3.93 kg/m^2^. As well, there were 154 males (61.1%) and 98 females (38.9%). The most common CAD risk factor was HTN (144 patients, 57.1%), followed by HLP (114 patients, 45.2%), DM (94 patients, 37.3%), smoking (75 patients, 29.8%), and FH (54 patients, 21.4%). Cardiac TnI was also at the elevated levels in 94 patients (37.3%), and the mean LVEF was 51.47 ± 6.15%. The patients were thus divided into two groups based on their echocardiographic E/(e′s′) ratio, including individuals with a low ratio (< 1.63) and those with a high score (≥ 1.63). The demographic and laboratory characteristics of both study groups are presented in Table 1. The patients with a high ratio were older and had a higher prevalence of female sex, as well as the SYNTAX score ≥ 22 than those with a low ratio (*p*-value < 0.001). Moreover, these patients had a lower GFR and a higher SYNTAX score than the group with a low score (*p*-value < 0.001 and 0.01, respectively). The echocardiographic characteristics of the study population are illustrated in Table 2. Among the echocardiographic variables, the indexed LA volume was larger, the E wave and E/e′ ratio were higher, and the LVEF, e′, and s′ velocities were lower in the patients with a high score than the individuals with a low value (*p*-value: 0.028, 0.004, < 0.001, 0.023, < 0.001 and < 0.001, respectively).

**Table 1:** Demographic, paraclinical, and angiographic variables of the two study subgroups characterized by high (≥ 1.63) and low (< 1.63) tissue-Doppler derived E / (e**′**s**′**) ratio

Variables	E/e**′**s**′** groups		*p*-value
	< 1.63 (n = 164)	≥ 1.63 (n = 88)	
Age (years)	57.4 ± 10.1	62.7 ± 9.0	< 0.001
*Sex, n (%)*			< 0.001
Male	114 (69.5%)	40 (45.5%)	
Female	50 (30.5%)	48 (54.5%)	
BMI (kg/m^2^)	27.2 ± 4.0	27.0 ± 3.8	0.646
HTN, n (%)	87 (53.0%)	57 (64.8%)	0.073
DM, n (%)	59 (36.0%)	35 (39.8%)	0.552
HLP, n (%)	74 (45.1%)	40 (45.5%)	0.960
FH, n (%)	37 (22.6%)	17 (19.3%)	0.550
Smoking, n (%)	53 (32.3%)	22 (25.0%)	0.226
GFR (ml/min)	89.0 ± 22.8	77.1 ± 22.1	< 0.001
Elevated TnI	100 (61.0%)	58 (65.9%)	0.440
SYNTAX score	21.0 ± 13.0	28.0 ± 11.5	< 0.001
SYNTAX score ≥ 22	88 (53.7%)	62 (70.5%)	0.01

Data are presented as mean ± SD or n (%)

BMI, Body mass index; DM, Diabetes mellitus; HTN, Hypertension; HLP, Hyperlipidemia; FH, Family history; TnI, Troponin-I; GFR, Glomerular filtration rate

**Table 2:** Echocardiographic variables of the two study subgroups characterized by high (≥ 1.63) and low (< 1.63) tissue-Doppler derived E / (e**′**s**′**) ratio

Variables	E/e′s′ groups		*p*-value
	< 1.63 (n = 164)	≥ 1.63 (n = 88)	
LVEF (%)	52.1 ± 5.9	50.2 ± 6.5	0.023
Indexed LA volume (ml/m^2^)	20.0 ± 6.9	22.1 ± 6.7	0.028
E wave (m/s)	0.60 ± 0.2	0.7 ± 0.2	0.004
A wave (m/s)	0.7 ± 0.18	0.7 ± 0.20	0.220
E/A	1.0 ± 0.9	1.3 ± 1.6	0.097
e′ (cm/s)	7.9 ± 2.0	5.1 ± 1.1	< 0.001
s′ (cm/s)	8.3 ± 1.5	5.8 ± 1.2	< 0.001
E/e′	8.0 ± 2.4	13.5 ± 3.6	< 0.001

Data are presented as mean ± standard deviation

LVEF, Left ventricular ejection fraction; LA, Left atrium; E wave, Transmitral Doppler early diastolic velocity; A wave, Transmitral Doppler late diastolic velocity; e′ velocity, Mitral annular early diastolic velocity; s′ velocity, Mitral annular peak systolic velocity

To determine the independent correlation between different variables and the coronary atherosclerosis severity explained by the SYNTX score, all variables were separately analyzed by univariate linear regression analysis. The variables with a *p*-value < 0.3 were thus imported into multiple linear regression via backward elimination (Table 3). The analysis outcomes then showed a positive independent association between age (B = 0.193, 95% CI 0.026–0.361, *p*-value = 0.024), the indexed LA volume (B = 0.244, 95% CI 0.006–0.481, *p*-value = 0.024), the positive TnI level (B = 3.988, 95% CI 0.744–7.232, *p*-value = 0.016), and the echocardiographic E/(e′s′) ratio ≥ 1.63 (B = 5.609, 95% CI 2.324–8.894, *p*-value = 0.001) and the SYNTAX score. To note, the E/e′ and E/(e′s′) ratios were not inserted together into the same multiple linear regression model, because they were closely related to each other and had a co-linear correlation (r = 0.87, *p*-value < 0.001). Therefore, the log transformation model was used, so that Ln (E/e′s′) was considered to be the sum of Ln (E/e′) and Ln (1/s′). This analysis revealed that Ln (1/s′) alone was not an independent predictor of the severity of the SYNTAX score (Table 4).

**Table 3:** Multiple linear regression analysis with backward elimination method determining association between different variables with SYNTAX score

Model		Unstandardized coefficients	Standardized coefficients	*p* value	95.0% Confidence interval for B	
		B	Beta		Lower bound	Upper bound
1	(Constant)	− 4.7		0.479	− 17.9	8.4
	Indexed LA volume	0.2	0.1	0.055	− 0.0	0.5
	age	0.2	0.1	0.061	− 0.0	0.3
	HTN	− 1.4	− 0.1	0.484	− 5.4	2.6
	HLP	− 1.3	− 0.1	0.497	− 5.2	2.5
	A wave	6.4	0.1	0.135	− 2.0	14.8
	E/e′s′ ≥ 1.63	5.7	0.2	0.001	2.4	9.0
	TnI	4.3	0.2	.011	1.0	7.5
3	( Constant)	− 10.6		0.049	− 21.2	− 0.0
	Indexed LA volume	0.2	0.1	0.044	0.0	0.5
	age	0.2	0.2	0.024	0.0	0.4
	A wave	7.0	0.1	0.096	− 1.3	15.2
	E/e′s′ ≥ 1.63	5.6	0.2	0.001	2.3	8.9
	TnI	4.0	0.2	0.016	0.7	7.2

LA, Left atrium; HTN, Hypertension; HLP, Hyperlipidemia; TnI, Troponin-I; E wave, Transmitral Doppler early diastolic velocity; A wave, Transmitral Doppler late diastolic velocity; e′ velocity, Mitral annular early diastolic velocity; s′ velocity, Mitral annular peak systolic velocity

**Table 4:** Log-transformation model based on final step of multiple linear regression analysis determining relationship between different constituents of E / (e**′**s**′**) ratio with SYNTAX score

Model		Unstandardized coefficients	Standardized coefficients	*P* value	95.0% Confidence interval for B	
		B	Beta		Lower bound	Upper bound
1	(Constant)	− 18.4		0.078	− 38.8	2.1
	Indexed LA volume	0.2	0.1	0.056	− 0.0	0.5
	age	0.3	0.2	0.001	0.1	0.4
	TnI	3.6	0.1	0.029	0.4	6.8
	Ln1/s′	− 2.2	− 0.0	0.771	− 17.0	12.6
	Ln E/e′	14.8	0.2	0.001	6.3	23.2

Dependent variable, syntax score

LA, Left atrium; TnI, Troponin-I; E wave, Transmitral Doppler early diastolic velocity; e′ velocity, Mitral annular early diastolic velocity; s′ velocity, Mitral annular peak systolic velocity

## Discussion

This study showed that the patients with NSTE-ACS and the E/(e′s′) ratio ≥ 1.63 were older and had a higher prevalence of female sex, as well as the SYNTAX score ≥ 22 and a lower GFR than those with a low ratio. Moreover, the indexed LA volume was larger, the E wave and the E/e′ ratio were higher, and the LVEF, e′, and s′ velocities were lower in the patients with a high score than the individuals with a low value.

Notably, the differential diagnosis of acute chest pain is wide, and involves some frequent life-threatening conditions, such as acute coronary syndrome and pulmonary embolism. Some novel markers have been accordingly developed to assess the severity and prognosis of acute chest pain syndromes. For example, the platelet-to-hemoglobin ratio (PHR) values had aided in predicting massive acute pulmonary emboli (APE), as an independent predictor of mortality in this respect [25]. On the other hand, the SYNTAX score is an anatomical scoring system which is often used to estimate the CAD severity based on the complexity, location, and functional significance of the coronary stenosis. However, studies on this subject have revealed the paucity of anatomical evaluation alone in determining the CAD complexity. Indeed, the efficiency of risk stratification should be improved by adding clinical and echocardiographic risk factors [26]. The prognosis of patients with NSTE-ACS is thus determined by myocardial injury and LV filling pressure. The LA size is also directly related to the diastolic LV filling pressure. The LA size, LV volume indices, and LVEF are accordingly the strong predictors of cardiovascular outcomes in patients with ACS. In the present study, the patients with the E/(e′s′) ratio ≥ 1.63 had a larger LA volume, a higher E wave velocity, and a lower LVEF than those with a lower ratio. As high E wave is associated with an elevated LA pressure, pulsed TD imaging is generally practiced to evaluate cardiac function, as an easily accessible and strongly reproducible modality. Contrary to previous methods of measuring the LV volumes and the LVEF, this new modality does not need to trace endocardial contours [1]. A high E/e′ ratio in patients with ACS is thus a marker of elevated LA pressure, and a strong predictor of adverse outcomes [8]. However, the changes in the LV preload can alter both E and e′ velocities, and some recent meta-analyses have further indicated that E/e′ is not helpful for the diagnosis of HF with preserved LVEF [27]. On the other hand, relaxation cannot be simply separated from contraction, but they should be considered together as the components of a continuous cycle. Recent studies have accordingly suggested the E/(e′s′) ratio as a powerful predictor of adverse outcomes in several cardiac illnesses, irrespective of the LVEF [28]. Indeed, a perfusion defect induces an increase in collagen turnover by stimulating ventricular remodeling. As a result, ischemia causes subclinical changes in the LV systolic or diastolic dysfunction, and the measurement of the e′ and s′ velocities can provide indirect information about myocardial perfusion. In this regard, Tiryakioglu et al. (29) conducted a prospective study on 111 patients presenting with acute anterior MI who had undergone successful PCI. An echocardiography was also performed in the first 24–36 h of admission and 6 months later. They further considered an increase of more than 20% in the diastolic volume and/or the systolic one as the LV remodeling. The patients were also divided into two groups, viz., those with and without LV remodelling. The initial septal E/(e′s′) values were significantly higher in the patients with the LV remodeling. Their proposed cutoff point for the E/(e′s′) ratio was 2.34 with 87.0% sensitivity and 82.1% specificity [29].

In 2021, Ionac et al. evaluated the prognostic value of the E/(e′s′) ratio in 307 consecutively hospitalized patients with NSTE-ACS and successful PCI, before discharge and six weeks later. The primary endpoint was thus considered as cardiac death or readmission due to re-infarction or HF. The results of the receiver operating characteristic (ROC) analysis also showed that the pre-discharge E/(e′s′) ratio was an excellent independent predictor of the composite outcome. The optimal cut-off value was further found to be 1.63 (namely, 74% sensitivity and 67% specificity) [1]. The SYNTAX score to measure the atherosclerosis severity was not used in this study. Based on the proposed cut-off point in the aforementioned study, our patients were divided into two groups with those scoring on one side of the cut-off value. As well, the patients were divided into two groups, according to their SYNTAX scores with the cut-offs used in the American/European guidelines. Accordingly, they were classified as low (≤ 22) and high scores (> 22). Of note, the high SYNTAX score is an independent predictor of a more complex and extensive CAD and the need for an early invasive strategy [30].

Old age has been further introduced as an important predictor of increased hospital mortality among different types of ACS. Old patients with ACS usually experience a complicated course, and are more prone to developing HF [31]. In our study, the patients with the higher E/(e′s′) ratio were older than those with the lower ratio. Compared with men, women with NSTE-ACS typically have an older age, have multiple cardiovascular risk factors, and are under treatment. They also have higher rates of myocardial ischemia and significantly higher all-cause mortality [32]. On the other hand, medically managed ACS patients with worse chronic kidney disease (CKD) show markedly increased long-term risks of ischemic and bleeding complications [33]. These findings were consistent with those in our study in which the patients with the higher E/(e′s′) ratio had a higher female prevalence and a lower GFR than those with the lower ratio. To the best of the authors’ knowledge, this study was the first attempt to evaluate the association between the E/(e′s′) ratio ≥ 1.63 and the SYNTAX score in the patients hospitalized with NSTE-ACS.

## Conclusion

The study results demonstrated that the patients hospitalized with NSTE-ACS and the E/(e′s′) ratio ≥ 1.63 had worse demographic, echocardiographic, and laboratory profiles and a higher prevalence of a SYNTAX score ≥ 22 than those with the lower ratio. Among different demographic, laboratory, and echocardiographic variables, there was an independent association between the E/(e′s′) ratio ≥ 1.63 and the SYNTAX score.

## Limitations

As the first limitation, a single-center descriptive analytical research design was used in this study on a small sample of patients with NSTE-ACS, admitted within a specified time period, who were not followed-up. In addition, more sophisticated and newer echocardiography modalities, such as speckle tracking echocardiography (STE), were not implemented in this study, which can be assumed as the limitation and strength. Although strain imaging seems to be more sensitive for determining the subtle changes of both systolic and diastolic functions than TD imaging, there were attempts to find an easily accessible and rapid way for bedside assessment, and stratify patients with NSTE-ACS into low- and high-risk groups.

## Data Availability

The datasets used during this research are not publicly available because of privacy and ethical restrictions. However, they are available from the corresponding author on reasonable request.

## Abbreviations

NSTE-ACS: Non-ST elevation-acute coronary syndrome
HF: Heart failure
LV: Left ventricle
TDI: Tissue Doppler imaging
s′: Mitral annular peak systolic velocity
e′: Early diastolic velocity
E wave: Early transmitral flow velocity
A wave: Transmitral Doppler late diastolic velocity
LVEF: LV ejection fraction
CAD: Coronary artery disease
ACS: Acute coronary syndrome
MI: Myocardial infarction
TnI: Troponin I
LBBB: Left bundle branch block
PCI: Percutaneous coronary intervention
CAG: Coronary angiography
HTN: Hypertension
DM: Diabetes mellitus
HLP: Hyperlipidemia
FH: Family history
BMI: Body mass index
GFR: Glomerular filtration rate
LVESV: Left ventricular end-systolic volume
LVEDV: Left ventricular end-diastolic volume
LA: Left atrium
ROC: Receiver operating characteristic
